# Dll4/Notch1 signalling pathway is required in collective invasion of salivary adenoid cystic carcinoma

**DOI:** 10.3892/or.2022.8268

**Published:** 2022-01-19

**Authors:** Ke Wang, Hua-Yang Fan, Xing Pang, Mei Zhang, Xiang-Hua Yu, Jia-Shun Wu, Bing-Jun Chen, Jian Jiang, Xin-Hua Liang, Ya-Ling Tang

Oncol Rep 45: 1011-1022, 2021; DOI: 10.3892/or.2021.7939

Following the publication of this article, the authors have realized that they made an error during the compilation of the images shown in Fig. 6, and that this error was not corrected before the paper was sent to press. Specifically, in Fig. 6B, the data panels showing the results from the HUVEC + SACC-83 si-Dll4 and HUVEC + SACC-LM si-Dll4 experiments at 24 h were inadvertently repeated.

The corrected version of Fig. 6, showing the correctly assembled data panels for Fig. 6B, is shown on the next page. The authors sincerely apologize for the errors that were introduced during the preparation of this Figure, thank the Editor for allowing them the opportunity to publish this Corrigendum, and regret any inconvenience that these errors may have caused.

## Figures and Tables

**Figure 6.**
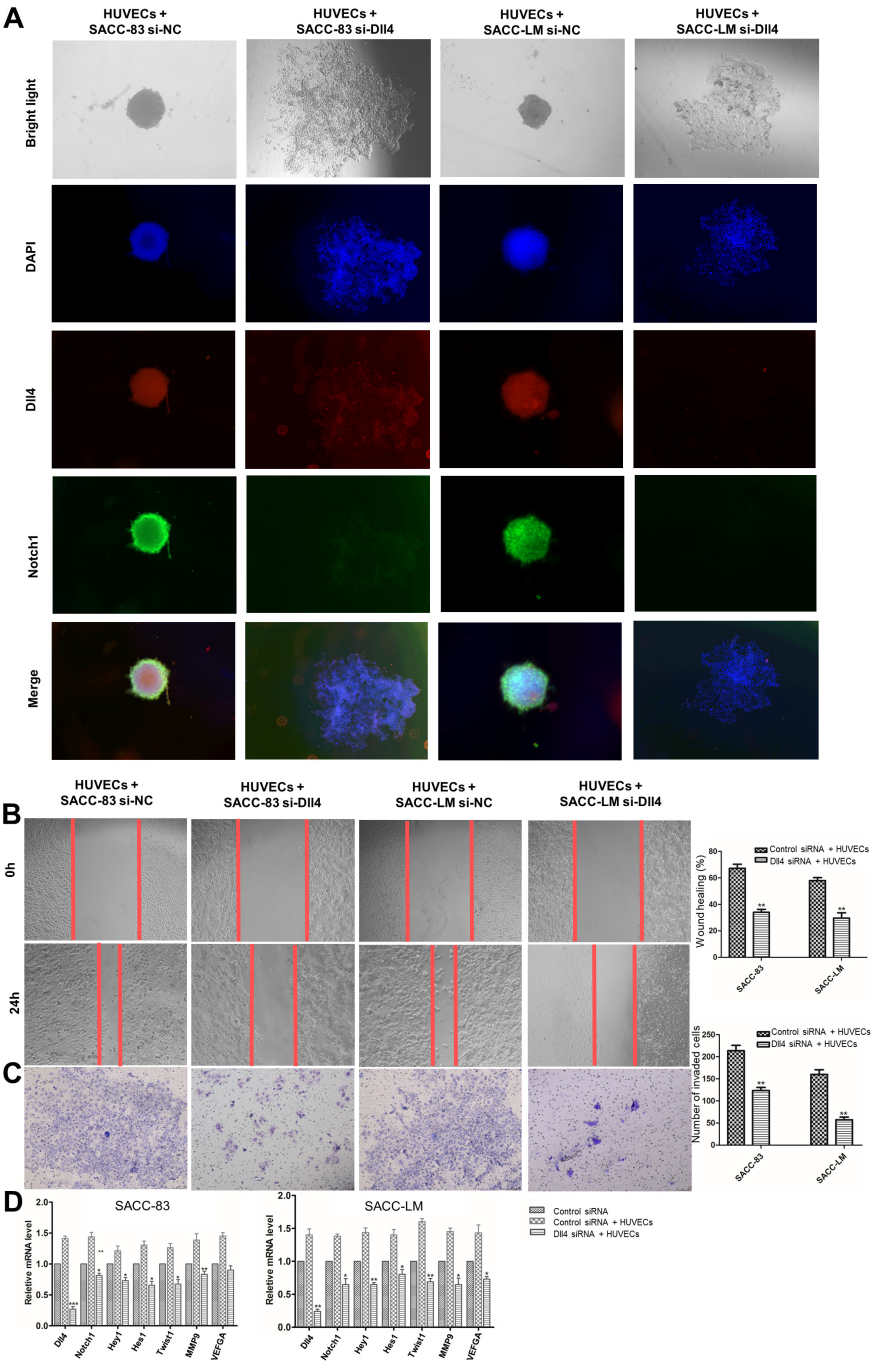
HUVECs regulates SACC collective invasion via the Dll4/Notch1 signalling pathway. (A) Immunofluorescence results for Dll4 expression (red) and Notch1 expression (green) in SACC cells transfected with Dll4 siRNA-3 or control siRNA during co-culture with HUVECs (magnification, ×40). (B) The migration of SACC cells transfected with Dll4 siRNA-3 or control siRNA during co-culture with HUVECs (magnification, ×100). (C) The invasion of SACC cells transfected with Dll4 siRNA-3 or control siRNA during co-culture with HUVECs (magnification, ×100). (D) The relative mRNA expression levels of Dll4, Notch1, Hey1, Hes1, Twist1, MMP9 and VEGFA in SACC cells transfected with Dll4 siRNA-3 or control siRNA during co-culture with HUVECs. The data are presented as the mean ± standard deviation (n=3). ^*^P<0.05, ^**^P<0.01, ^***^P<0.001 vs. control siRNA + HUVECs. SACC, salivary adenoid cystic carcinoma; Dll4, δ-like ligand 4; siRNA, small interfering RNA; HUVECs, human umbilical vein endothelial cells; Hey1, hairy/enhancer-of-split related with YRPW motif protein 1; Hes1, transcription factor HES-1; Twist1, Twist-related protein 1.

